# Sensory reliability takes priority over the central tendency effect in temporal and spatial estimation

**DOI:** 10.1038/s41598-025-22651-6

**Published:** 2025-11-06

**Authors:** Alessia Tonelli, Cameron K. Phan, David Alais

**Affiliations:** 1 School of Psychology, The University of Sydney, Sydney, Australia; 2 Present Address: UVIP - Unit for Visually Impaired People, Fondazione Istituto Italiano di Tecnologia, Genoa, Italy

**Keywords:** Time perception, Spatial perception, Central tendency, Auditory perception, Visual perception, Auditory system, Sensory processing, Visual system

## Abstract

Perception is influenced by contextual factors that help resolve sensory uncertainty. A well-known phenomenon, the central tendency effect, describes how perceptual estimates gravitate toward the mean of a distribution of stimuli, particularly when sensory input is unreliable. However, in multisensory contexts, it remains unclear whether this effect follows a generalized priority across modalities or might be influenced by task-relevant sensory dominance. We studied spatial and temporal estimation in the auditory and visual modalities, testing whether perceptual estimates are driven by a supra-modal prior or by modality reliability specific to the task, and applied Bayesian modeling to explain the results. Participants first performed baseline sessions using only one modality and then a third session in which the modalities were interleaved. In the interleaved session, we found that the changes in auditory and visual estimates were not towards a supra-modal (generalized) prior, but estimates related to the dominant modality (vision for space, audition for time) were stable, while estimates of the other sensory modality (audition for space, vision for time) were pulled towards the dominant modality’s prior. Bayesian modeling also confirmed that the best-fitting models were those in which priors were modality-specific rather than supra-modal. These results highlight that perceptual estimation favors sensory reliability over a general tendency to regress toward the mean, providing insights into how the brain integrates contextual information across modalities.

## Introduction

What we perceive often does not align perfectly with external reality because the brain has developed the ability to exploit contextual information to optimize perception. Adaptation^1^ and serial dependence^2^ are examples of how context can shape perception, as is the phenomenon of central tendency (or regression toward the mean). When exposed to a set of magnitudes, people tend to overestimate the lowest and underestimate the highest values so that the perceived value tends to gravitate toward the arithmetic mean of the set. The central tendency effect has been observed across a wide range of perceptual domains^3–7^ and indicates that the perception of a given stimulus is sensitive to the global context within which it occurs. This phenomenon can be explained within the Bayesian framework in which the brain combines incoming sensory inputs with prior knowledge to generate optimal world estimates^8^.

According to this idea, the brain uses the central tendency effect to handle the uncertainty associated with perceptual information, where signal variability is often associated with noise rather than real variation of the physical stimulus. For this reason, to resolve the ambiguity inherent in the task, people need to use additional information, such as that provided by context, to maximize perceptual precision. If perceptual information is highly reliable, the contextual information is down-weighted, resulting in a more veridical perception.

Related to this, studies on multisensory perception have shown that sensory sensitivity and sensory ambiguity vary according to the task at hand^9^. In particular, hearing is considered the most reliable sense in temporal perception tasks^10,11^, while vision is more reliable for spatial perception tasks^12,13^. When visual and auditory information conflict, our perception is shifted toward the most reliable sensory modality, as in the case of the ventriloquist effect^14^. In a purely temporal context, our perception will be “captured” by sound^15,16^, while in a spatial context, it will be “captured” by vision^12,17^. In addition, McGovern et al.^18^ found that perceptual learning is transferable between modalities but follows a similar principle: spatial training in the visual modality is transferred to audition but not vice versa, and conversely, temporal training in the auditory modality transfers to vision but not vice versa.

Therefore, there are contexts that accrue over time, such as the central tendency effect generated by exposure to a set of values (supra-modal context), and contexts that arise instantaneously based on task requirements, such as an auditory bias upon exposure to a temporal task (sensory context). What happens to our perception if both contexts exist – will they interact, or are they separate and non-interacting? To test this, we measured temporal and spatial perception using an estimation task and compared performance for auditory and visual stimuli. This manipulates sensory priority through task context, as vision is more reliable for spatial tasks and audition for temporal tasks. Furthermore, we assigned each sensory modality a different stimulus range (durations or distances). This allowed us to test whether separate central tendency effects are formed within the same session (each modality converging to a different mean) or whether a global (supra-modal) prior emerges that converges on the mean of both stimulus ranges. In separate sessions, participants estimated the temporal or the spatial interval of an auditory or visual stimulus to provide baseline performance. This captures the central tendency effects for each sensory modality separately because the global and sensory-specific priors will be equivalent when only one sense is tested at a time. In a third interleaved A-V session, auditory and visual trials were randomly intermixed. This is a pivotal session because stimulus estimation can potentially be affected by three competing priors, allowing a test of whether perception is affected by sensory-specific central tendency effects or by a single supra-modal effect. In the case of sensory-specific central tendency, we would expect perception to be influenced by the prior of the most reliable modality for a given task (audition for time, vision for space), while the less reliable modality will show a bias toward the prior of the dominant one. Conversely, if perception is influenced by a supra-modal prior, estimates from both modalities should regress toward a single global prior computed from the joint stimulus distribution.

In this context, we define modality-specific priors as expectations learned from the statistical structure of a single sensory modality (e.g., auditory durations or visual lengths). These priors are rooted in the modality from which they derive, but under conditions of strong sensory dominance, as induced by our experimental design, they can influence perception across modalities in a “winner-takes-all” manner. For example, in the temporal task, although participants estimated durations in both auditory and visual trials, their responses were biased toward the auditory prior, reflecting the greater reliability of hearing in that context.

In contrast, we use the term “supra-modal prior” to refer to expectations encoded in a modality-independent fashion, abstracted from any single sensory input. Such priors would influence perception across modalities regardless of which modality is dominant, reflecting a shared, central representation of stimulus statistics.

The data were evaluated for both central tendency and sensory context effects. To formally assess these hypotheses, we compared the predictions from four Bayesian models with different assumptions about the prior. Overall, results indicate that perceptual estimation is driven by sensory dominance, meaning that sensory-specific context governs temporal and spatial estimation more than supra-modal central tendency.

## Results

### Behavioral data

**Fig. 1:**
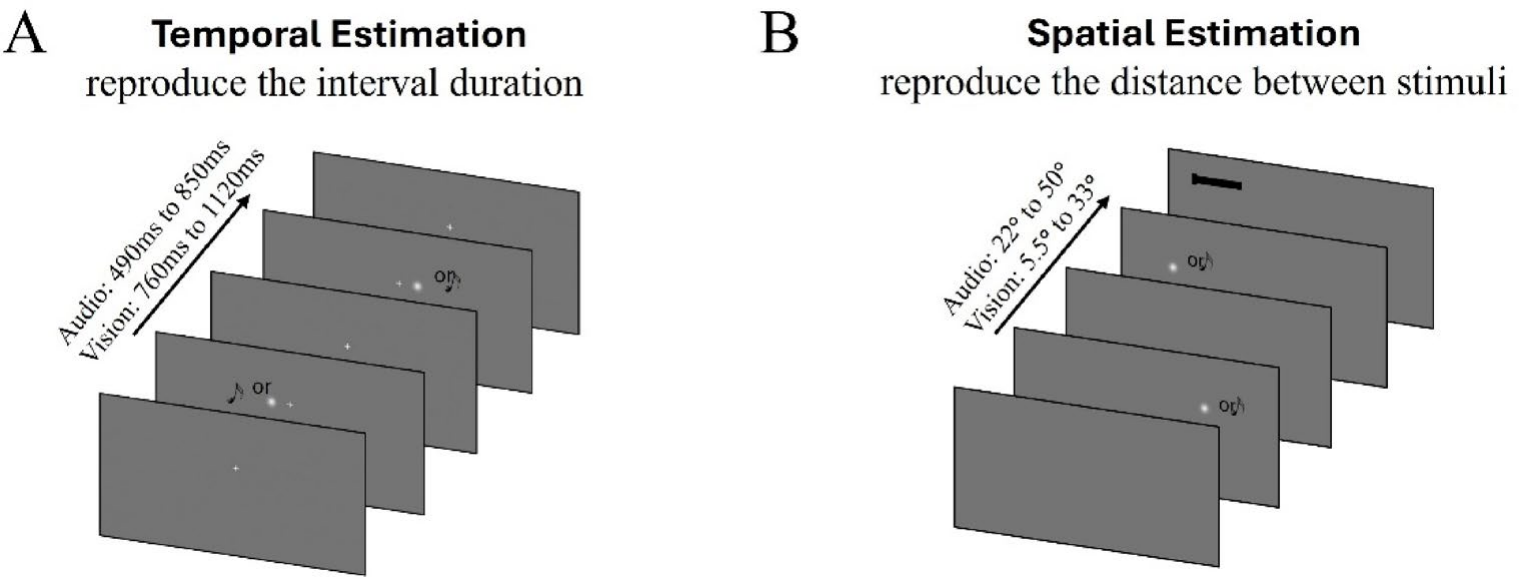
Experimental paradigms. (**A**) Temporal estimation task: participants reproduce the interval defined by two successive stimuli (visual or auditory) by holding a mouse button. (**B**) Temporal estimation task: Two successive stimuli define a spatial interval, and participants use a key-controlled slider to reproduce the spatial extent.

All participants performed the two tasks shown in Fig. 1: temporal estimation, and spatial estimation. Each task was done in an auditory and a visual version. For the two estimation tasks, we, first, considered the baseline sessions for which we calculated the root mean squared error (RMSE) to obtain an overall performance and then the average sensory uncertainty for each modality by calculating the weight assigned to it, which is inversely proportional to its variance (see the Data Analysis section). Moreover, for all sessions, we quantified the slope of the linear fit between the actual and perceived stimuli and the y-intercept. As shown in Fig. 3, a slope < 1 indicates that perceived values regress to the mean of the stimulus set (a slope of 1 indicates no regression). Therefore, we define a Regression Index (RI) to quantify the central tendency effect as one minus the slope so that RI = 0 means perception is veridical and values > 0 indicate regression to the mean (with 1.0 indicating complete regression to the mean). The RI can verify whether there is a sensory domain-specific to the task. Moreover, in the interleaved A-V session, we use the RI to determine whether perception is mainly influenced by the classical central tendency effect (supra-modal context) or instead follows the sensory context driven by the task sensory specificity (i.e., vision for spatial task and audition for temporal task), whereby stimuli from the non-dominant modality are biased in the direction of the mean of stimuli from the dominant one. To quantify this bias, we used the y-intercept obtained from the linear fit to the data.

**Fig. 2:**
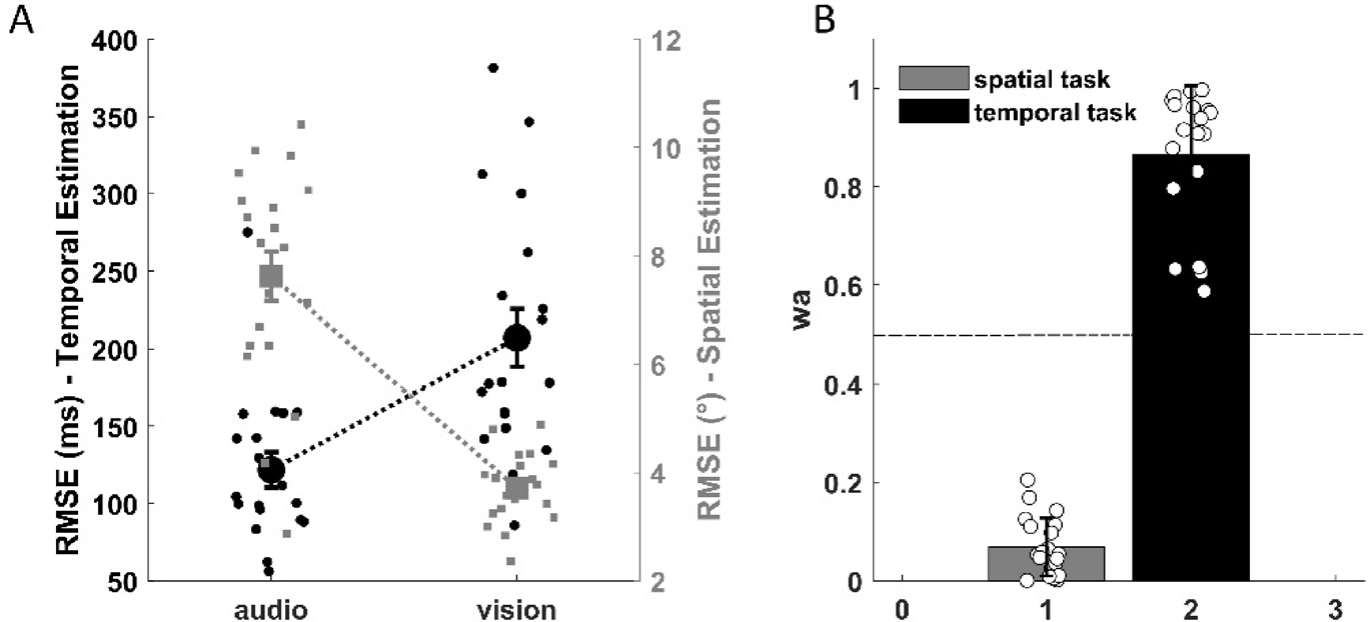
(**A**) Data for the estimation task showing average RMSE (i.e., overall performance) for the temporal (in black) and spatial (in grey) tasks for each sensory modality. (**B**) Average weight given to the auditory modality for the temporal (in black) and spatial (in grey) tasks (wv = 1-wa). In both plots, small squares and circles represent individual performance. Large symbols are group means with the error bars showing ± 1 s.e.m.

Data for the spatial and temporal estimation tasks (Fig. 2A) show a similar task-specific sensory dominance. Again, temporal estimation (in black) is better (lower RMSE) using the auditory modality than the visual (two-tail paired t-test: t(18) = 7.48, *p* < 0.0001, CI95% [-109.49, -61.49]), while in the spatial estimation task (in grey) the RMSE is lower using the visual modality than the auditory (two-tail paired t-test: t(19) = 8.01, *p* < 0.0001, CI95% [-4.93, -2.89]).

Moreover, in Fig. 2B, the average weights related to the auditory modality (w_a_) are represented. If the sensory modalities are weighed equally, the values should revolve around 0.5 (dashed line). Instead, we found that there is a clear imbalance between the sensory modalities according to the task: in the temporal task, the w_a_ is greater than 0.5 (thus the visual modality) - (one sample t-test against 0.5, t(18) = 11.37, *p* < 0.001, CI95% [0.798 0.933]) -, vice versa for the spatial task in which w_a_ is lower than 0.5 (one sample t-test against 0.5, t(19) = -32.54, *p* < 0.001, CI95% [0.041 0.096]).

**Fig. 3:**
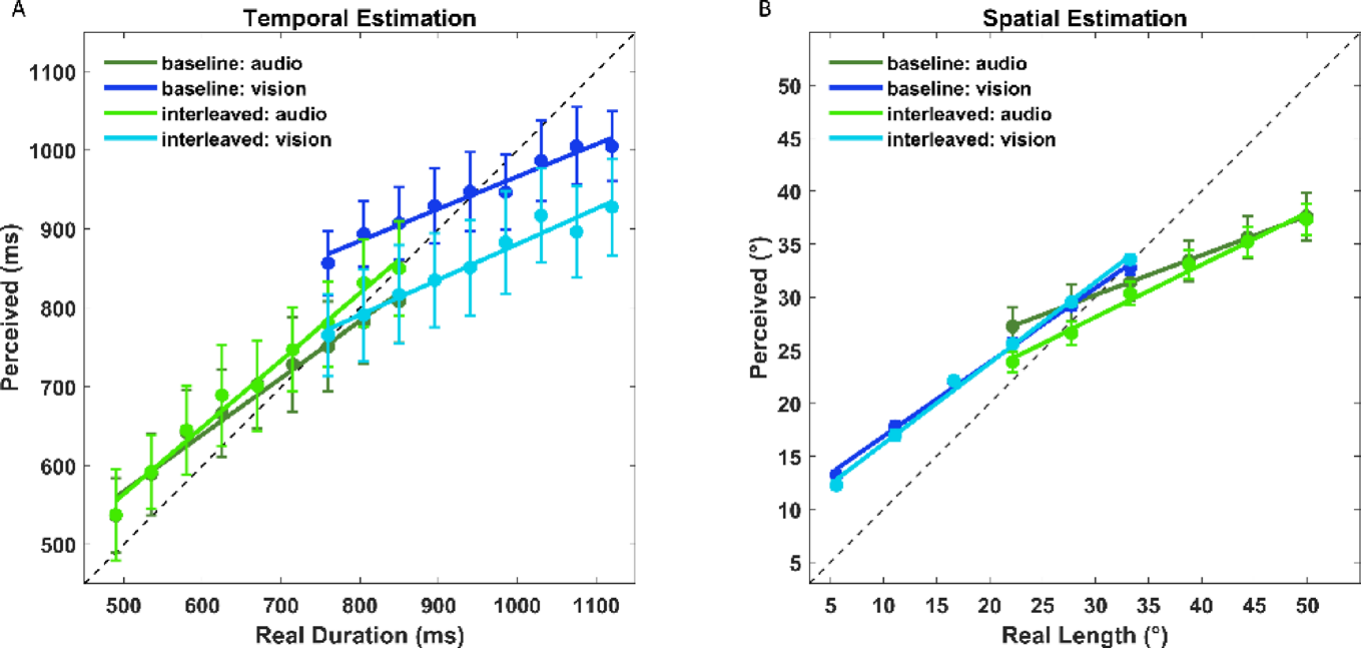
(**A**) Overview of the results for the temporal estimation task. Dark colors represent the average perceived durations in the baseline sessions for audio (dark green) and vision (blue). Instead, the lighter colors represent the average perceived durations in the interleaved session and are plotted considering auditory (light green) and visual (cyan) trials. (**B**) Overview of the results for the spatial estimation task. Dark colors represent the average perceived lengths for audio (dark green) and vision (blue) in the baseline sessions. Instead, the lighter colors represent the average perceived lengths in the interleaved A-V session and are plotted considering auditory (light green) and visual (cyan) trials. Error bars are the s.e.m.

Figure 3 plots stimulus estimations against actual stimuli for temporal (Fig. 3A) and spatial (Fig. 3B) estimation tasks, as obtained in auditory-only, visual-only, or interleaved A-V conditions. It is clear upon visual inspection that there is an effect of central tendency in all modalities and sessions (i.e., all slopes < 1). However, there is additional modulation in the interleaved A-V sessions compared to the baseline unimodal sessions for both the temporal and spatial tasks.

**Fig. 4:**
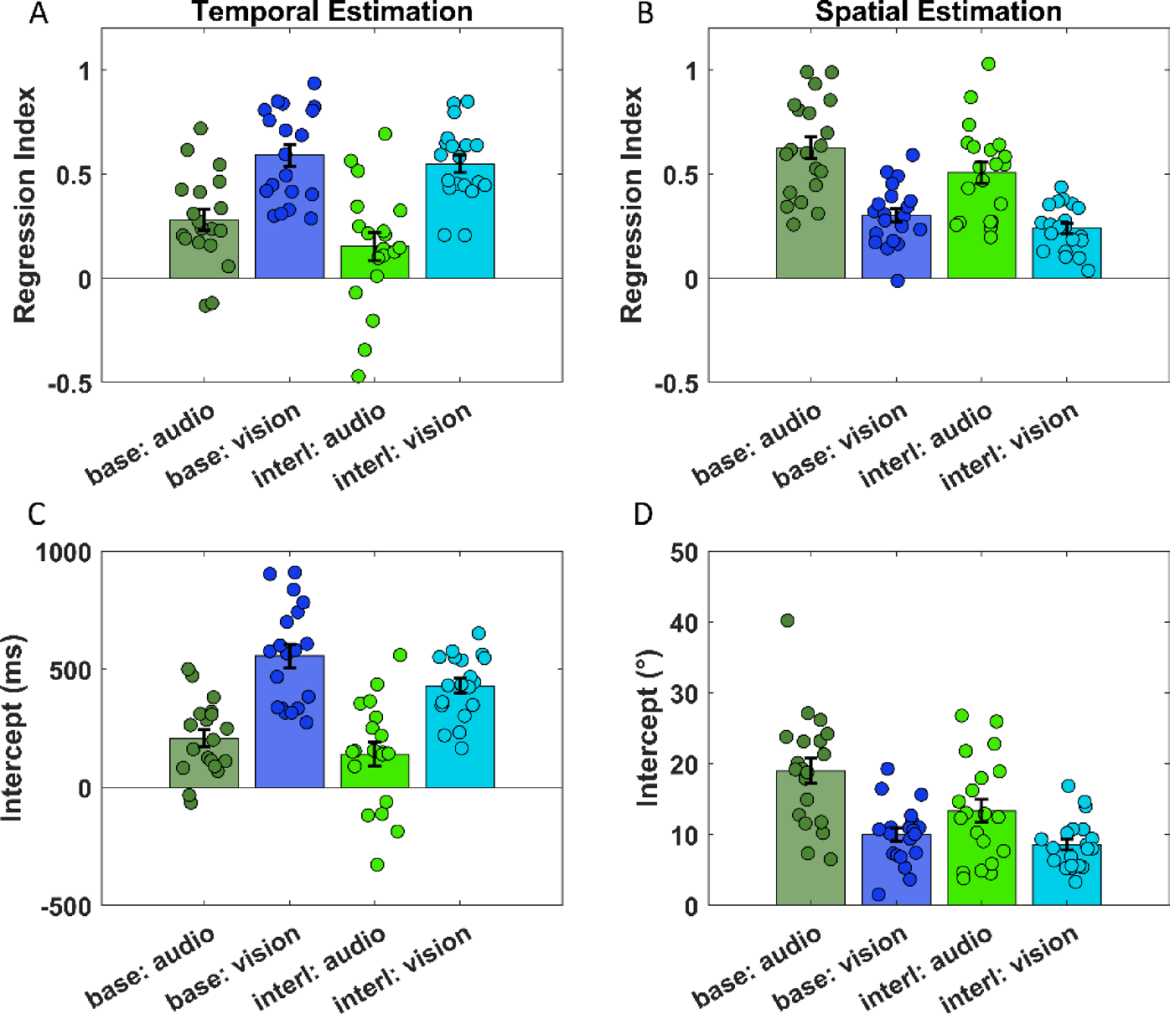
(**A**) Bar plots are the average regression index in the different sessions of the temporal estimation task divided for audio (shades of green) and vision (shades of blue). (**B**) Bar plots are the average regression index in the different sessions of the spatial estimation task divided for audio (shades of green) and vision (shades of blue). (**C**) Bar plots are the average intercept of the linear fit (beta) in the different sessions of the temporal estimation task divided for audio (shades of green) and vision (shades of blue). (**D**) Bar plots are the average intercept of the linear fit (beta) in the different sessions of the spatial estimation task divided for audio (shades of green) and vision (shades of blue). In all plots, circles represent the individual data, and the error bars represent the s.e.m.

Figure 4 plots data for the estimation tasks and shows the effects of both regression to the mean (upper panels) and intercept (lower panels), for temporal estimation (left panels) and spatial estimation (right panels). For the temporal task, we found a higher regression to the mean (Fig. 4A) for visual compared to the auditory stimuli in the single-modality sessions (t(54) = 4.93, *p* < 0.0001, Cohen’s d = 1.6), meaning that in the condition where sensory information for timing is noisier, there is a greater use of contextual information (i.e., higher RI). A similar difference remained (indeed, it is even more pronounced) if we compared the RIs for the two sensory modalities in the interleaved A-V session (t(54) = 6.33, *p* < 0.0001, Cohen’s d = 2.05). We did not observe a significant difference between the single-modality sessions and the interleaved A-V session, either for audio (t(54) = 2.06, *p* = 0.09, Cohen’s d = 0.67) or vision (t(54) = 0.66, *p* = 0.515, Cohen’s d = 0.21). The fact that the difference between the two modalities in the interleaved A-V session is maintained and shows no change in RI between the single and interleaved sessions, even though the average prior between the sessions differs, could be interpreted as a reduced influence of central tendency effect in the interleaved A-V session. This might indicate that visual and auditory information are processed separately. However, this interpretation might not be supported, looking at y-intercept data from the linear fits that capture sensory bias (Fig. 4C), because while there is no change for the auditory modality between single-modality and interleaved A-V (t(54) = 1.3, *p* = 0.2, Cohen’s d = 0.42) sessions, this was not true for the visual modality. Indeed, in the interleaved A-V session, visual stimuli are underestimated compared with the single-modality session, causing a reduced y-intercept (t(54) = 2.43, *p* < 0.05, Cohen’s d = 0.79). Results for the spatial estimation task showed a complementary pattern. We found a higher regression to the mean for auditory compared to visual stimuli in the unimodal sessions (t(57) = 7.42, *p* < 0.0001, Cohen’s d = 2.35), as shown in Fig. 4B. This means that in the auditory condition, where sensory information is noisier for spatial tasks, there is greater use of contextual information and, thus, greater regression toward the mean. Also, in the interleaved A-V condition, the RI of the two sensory modalities differed (t(57) = 6.14, *p* < 0.0001, Cohen’s d = 1.94). Moreover, we did not observe a significant difference between the baseline session and the interleaved A-V session for vision (t (57) = 1.44, *p* = 0.15, Cohen’s d = 0.46), but there is a slight difference for audio in which in the interleaved A-V session the RI is lower (t (57) = 2.72, *p* < 0.05, Cohen’s d = 0.86). Nevertheless, if we look at the sensory bias results (i.e., y-intercepts shown in Fig. 4D), we found no change for the visual modality between unimodal and interleaved A-V sessions (t(57) = 0.87, *p* = 0.39, Cohen’s d = 0.28), but it changes for the auditory modality. Indeed, in the interleaved A-V session, auditory stimuli are underestimated compared with the single-modality session, causing a reduction in y-intercept (t (57) = 3.7, *p* < 0.01, Cohen’s d = 1.17). So, for the spatial estimation, we could speculate that a cross-modal effect is present in the interleaved session in which auditory perception is influenced more by the visual prior than by the mean of the distribution and, thus, a stronger sensory context.

### Bayesian model

The behavioral data showed that the impact of this prior, i.e., the distribution of the stimuli, is task-specific and sensory-specific. Indeed, we found that in the spatial task, the effect is significantly larger with the auditory modality. Conversely, the effect is more significant for the visual modality for temporal tasks because each modality corresponds to the least reliable modalities for the specific task.

In the interleaved A-V session, behavioral data suggests that the distribution of the stimuli (prior) to influence, especially the estimate of the least reliable modality (vision for time, hearing for space), is not the mean of the overall AV distribution, i.e., a supra-modal central tendency effect, but rather this estimate is affected more by the sensory context.

**Fig. 5:**
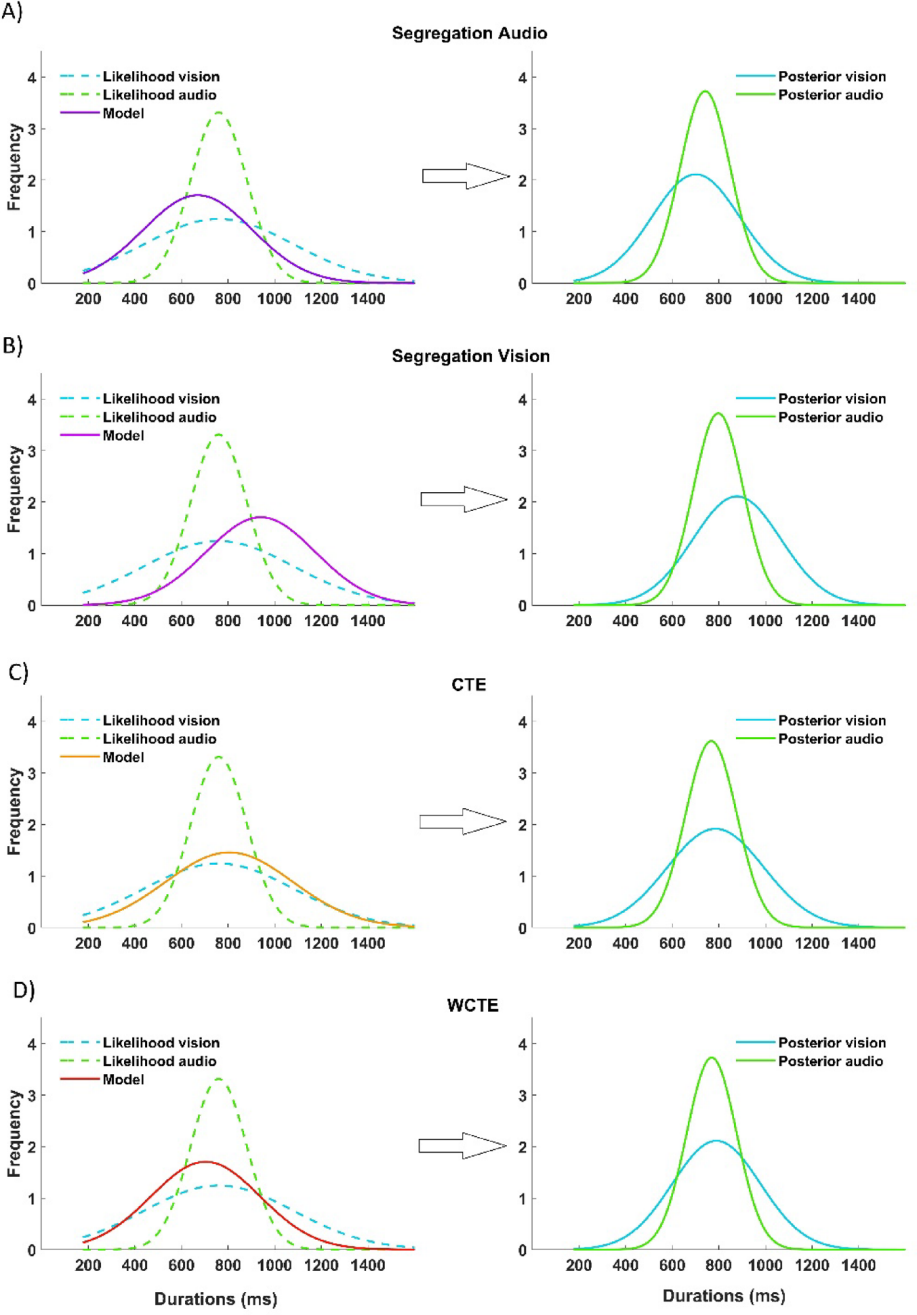
Schematic of different models, using temporal estimation as an example. For all the plots in the left column, the likelihoods (dashed lines) for audio (in green) and vision (in cyan) are modeled by a Gaussian distribution centered on the current stimulus to estimate and with a width corresponding to the sensory precision of each modality. In the specific case of temporal performance, the precision is higher for auditory stimuli (in green) than visual stimuli (in cyan). The prior is represented by the Gaussian distribution based on the assumption of each model. In the audio segregation model (**A**), the prior is centered on the average of the auditory stimuli only. In the vision segregation model (**B**), the prior is centered on the average visual stimuli only. In the central tendency effect model (**C**), the prior is centered on the average of the stimuli in the session, i.e., averaging both auditory and visual stimuli. In the weighted central tendency effect model (**D**), the prior is centered on the average of the stimuli in the session, taking into account the weight given to each sensory modality, i.e., averaging both auditory and visual stimuli. The posteriors are represented on the right column by the green and cyan continuous lines, respectively, for audio and vision, as a combination of prior and likelihood.

Within the Bayesian framework, the estimated stimulus (or posterior) is the combination of two sources of information: the *likelihood*, i.e., the current noisy estimate of stimulus durations/lengths represented by a Gaussian distribution centered at the current stimulus, and the *prior*, i.e., which reflects the observer’s internal expectation based on previously experienced stimuli. While the true stimulus distribution in our experiment is uniform (a boxcar function), it is not made explicitly available to the observer. Therefore, in line with previous literature^19,20^, we modeled the prior as a truncated normal distribution. Its parameters were estimated such that the expected value and variance of the prior matched those of the actual stimulus distribution, while respecting the physical bounds of the stimuli. An example of the model-based prediction of behavior is presented in Fig. 5 for the temporal estimation task.

To further verify if the behavioral data in the interleaved A-V session are influenced by a supra-modal central tendency effect, rather than the sensory context, we considered four models (described in Materials and Methods/Bayesian Models) mimicking the behavior of an ideal Bayesian observer (Fig. 5), manipulating the nature of the prior influencing the response. The ‘audio segregation’ model (Fig. 5A), in which the prior is centered on the average of the auditory stimuli only. The ‘vision segregation’ model (Fig. 5B), in which the prior is centered on the average of the visual stimuli only. The ‘central tendency’ model (Fig. 5C), in which the prior is centered on the average of the stimuli in the session, i.e., averaging both auditory and visual stimuli. Finally, the “weighted central tendency” model (Fig. 5D), in which the prior is centered on the average of the stimuli in the session, considering the weight given to each sensory modality, i.e., the addition of the product of the averages of both auditory and visual stimuli with their respective weight. Moreover, we assume that the *likelihood* (Fig. 5 dashed lines) varies according to the sensory modality. The location (µ) matches the stimulus to be estimated, while the width (σ) of the distribution corresponds to the sensory precision for that specific stimulus and sensory modality obtained from the baseline sessions (see data analysis section).

**Fig. 6:**
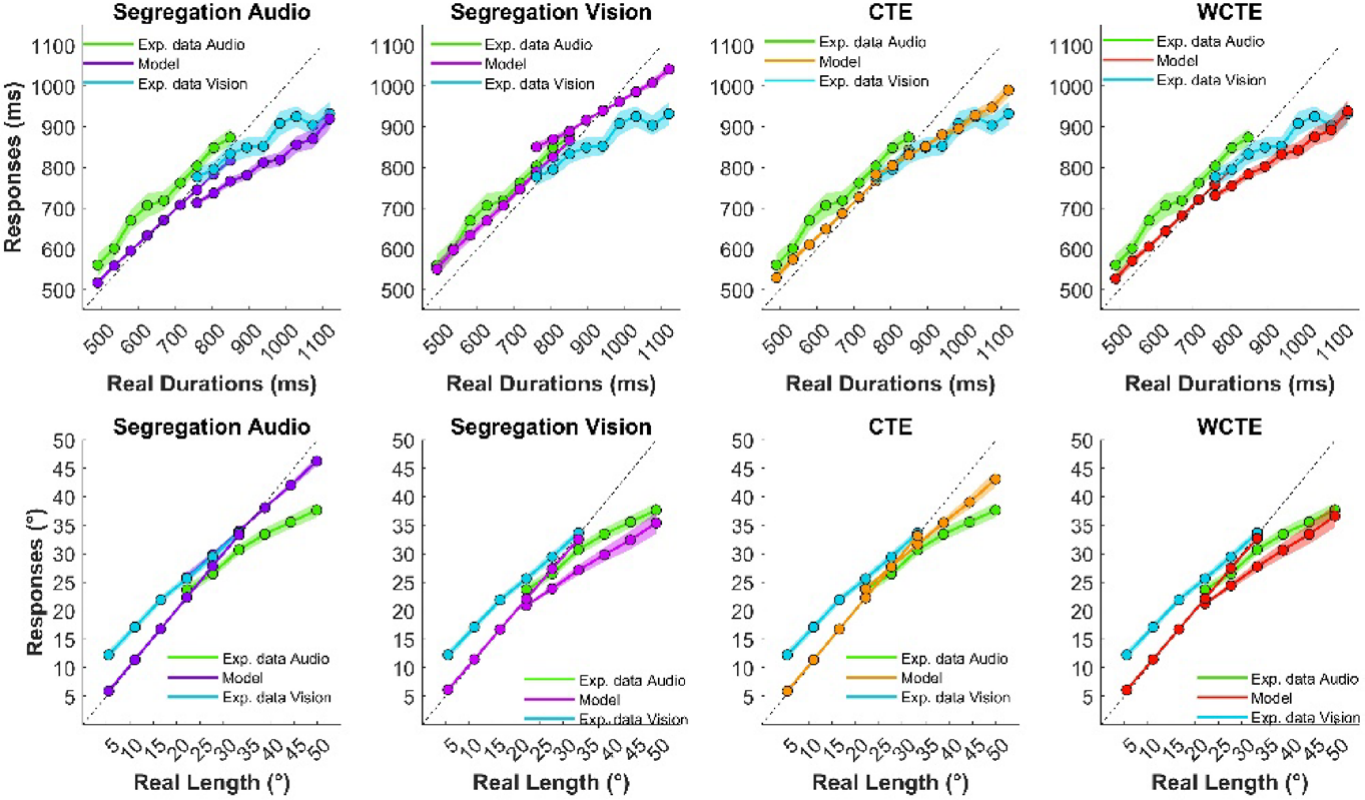
Comparison between the averaged real experimental data obtained in the interleaved session for audio (in green) and vision (in cyan) and the data simulated by each model. On the top row are the results of the temporal estimation, while on the bottom are the results of the spatial estimation.

By manipulating the nature of the *prior* distribution and, consequently, the posterior, we were able to see which of the four models best predicted the behavioral data and answer the question of whether context influenced the estimation, the supra-modal central tendency effect versus the sensory context. An example that considers temporal estimation is represented in Fig. 5. For all the plots, the likelihood is constant (dashed lines) and modeled by a Gaussian with a width corresponding to the sensory precision of each modality. In temporal performance, the precision is higher for auditory stimuli (in green) than visual stimuli (in cyan). The prior is represented by the Gaussian distribution based on the assumption of each model. Each plot shows how the posterior (green continuous line for audio and cyan continuous line for vision) is modulated by changing the prior. The outcomes of the models are represented in Fig. 6 on the top row for temporal estimation and on the bottom row for spatial estimation.

For temporal estimation, according to the statistical analysis, the best model for the experimental data is the segregation audio with an AIC of -755.067 and an R^2^ of 0.76 (see Table 1).

**Table 1:** Summary models comparison.

	A.I.C.	logLik	Deviance	*R* ^2^	Predicted *R*^2^
**Model for temporal estimation**					
SegAudio	− 755.07	383.53	− 767.07	0.76	0.73
SegVision	− 696.96	354.48	− 708.96	0.71	0.675
CTE	− 741.05	376.53	− 753.05	0.75	0.71
WCTE	− 754.38	383.19	− 766.38	0.76	0.729
**Model for spatial estimation**					
SegAudio	1302.03	− 645.02	1290	0.84	0.81
SegVision	1278.83	− 633.42	1266.8	0.865	0.84
CTE	1297.89	− 642.94	1285.9	0.85	0.81
WCTE	1281.2	− 634.6	1269.2	0.86	0.83

For spatial estimation, all the models replicate the visual trials quite well. Still, only the segregation vision and central tendency effect models mimic the estimation for the auditory trials. However, the statistical analysis indicates the segregation vision model is the best predictor, with an AIC of 1278 and an R^2^ of 0.865 (see Table 1).

These results support the idea that perceptual estimation is driven by sensory dominance, and therefore by sensory context, more than the supra-modal central tendency effect. However, it is worth noting that the results of the WCTE model are very similar to the segregation model, visual for the spatial task and auditory for the temporal task. In the temporal task, for example, the ΔAIC between the WCTE and the segregation-audio model was only 0.69, which falls within the range typically interpreted as substantial support for both models. This similarity likely reflects the strong imbalance in how the sensory modalities are weighted, i.e., the visual modality is weighted much more than the auditory modality in the spatial task, vice versa in the temporal task.

## Discussion

Creating prior expectations is essential for dealing with the complexity of an uncertain environment. Priors can be built over a lifetime through implicit learning from natural statistics and remain stable^21,22^ or can be context-dependent priors that are built quickly and are easy to manipulate^2,23,24^. An example of context-dependent priors is the central tendency effect^19^ investigated in the present study. Our first finding suggests that recent sensory experience builds up a central tendency effect that is not generalized but linked to the dominant modality for a specific task. In the interleaved A-V session, we found that the changes in auditory and visual estimates were not towards a supra-modal (generalized) prior, as expected by the “traditional” central tendency effect. Instead, estimates of stimuli related to the dominant modality (vision for space, audition for time) were stable, while estimates of the other sensory modality (audition for space, vision for time) were pulled towards the dominant modality’s prior. This pattern suggests dominance based on task-specific sensory reliability. These results are in line with previous studies on multisensory perception, showing that sensory reliability and ambiguity vary according to the task at hand^9^. This is evident when there is a conflict between modalities, in which one modality is weighted more than the other, leading to a bias toward one or the other modality^14,15,25^. In particular, hearing is considered the most reliable sense in temporal perception tasks^10,11^, while vision is more reliable for spatial perception tasks^12,13^.

A note should be added, namely, previous studies examining the phenomenon of central tendency in multiple modalities have focused mainly on temporal perception^26,27^. These have shown that prior experience influences perception independently of the type of sensory modality used, where the observer creates a generalized prior across stimuli. For example, in the presence of auditory and visual stimuli, two different priors are not made^28^, nor is there a cross-modal effect between the two^27^. However, although our conclusions here seem to suggest otherwise, they are not mutually exclusive. For example, in the case of Zimmermann & Cicchini^27^, a cross-modal influence was probably not found because, to have a strong context effect, they had to increase the uncertainty of both the visual and auditory stimuli. By doing so, the sensory dominance of the normally more reliable modality was lost, and consequently, so too was the imbalance in weighting the two sensory modalities.

The central tendency effect can be explained within the Bayesian framework, in which the brain combines sensory inputs with prior knowledge to generate optimal world estimates^8^. For this reason, we tested the hypothesis of a supra-modal versus sensory context for central tendency, generating predictions from four models manipulating the prior influencing the estimation within the Bayesian framework. As a second result, we found that the model best predicting our data was the “segregation model”, which was task-specific, and not the one in which the prior was set to the average of the entire stimulus distribution, regardless of sensory modality. The best model predicting the experimental data had a prior centered on the average of the auditory stimuli for the temporal task and a prior centered on the average of the visual stimuli for the spatial task. Thus, we see a cross-modal effect in which the dominant sensory modality for a given task overtakes the other, similar to reports in the aforementioned studies on multisensory. Moreover, we can conclude that this weighting process is a generalized mechanism because we found it in both temporal and spatial domains.

Nevertheless, how can we explain this sensory-context dominance effect? According to the Bayesian framework, the central tendency effect is used by the brain to handle the uncertainty associated with perceptual information. The estimate of a stimulus would originate from the combination of two sets of information: the likelihood function for the current stimulus that is modeled by a Gaussian centered on the current distance and with a width corresponding to participants’ sensory precision, and the prior that is represented by a Gaussian probability density function derived from past trials^3,19^. It has been suggested that these two components are mapped differently in the human brain, with the likelihood activating regions of the brain at an early stage of sensory processing and the prior activating specialized later brain areas including the putamen, amygdala, parts of the insula, and orbitofrontal cortex^29^. It has been hypothesized that the brain might use these specialized areas because the formation of priors requires both integration over time and input directly from sensory areas^29^. We hypothesize that during the creation of a prior, a kind of competition between sensory modalities is activated to provide information to these areas, and that the sensory modality with less uncertainty (i.e., the dominant modality) has a greater weight in shaping them. If this were the case, the incongruency caused by the different distributions of stimuli within the two sensory modalities would activate competition, with the sensory-dominated modality emerging as the winner in shaping the final prior. In animal models, it has been shown that less reliable sensory signals show reduced activity when they conflict with dominant signals, highlighting a competition between sensory modalities^30^. Moreover, fMRI studies have shown that the planum temporale (a region of the auditory cortex) reduces its activity when a dominant visual signal forces auditory perception to conform to the position of the visual signal^31,32^. This suggests a neural suppression mechanism in humans for the subordinate signal. Moreover, recent findings suggest that the weighting between prior and likelihood may reflect stable individual traits. Goodwin et al.^33^ demonstrated that Bayesian belief updating in perceptual tasks shows high temporal stability, reinforcing the idea that individual differences in sensory weighting are consistent over time.

Nonetheless, it should be emphasized that in our study, we created a scenario in which there was a strong imbalance between how sensory modalities are weighted. Recent evidence supports the idea that priors training and use may also depend on supra-modal predictive systems. For example, Sabio-Albert et al.^34^ showed that when auditory and visual stimuli are presented in parallel and have predictable structures, the learning and prediction of statistical regularities across sensory modalities are not entirely independent and may be mediated by a supra-modal predictive system. Although their paradigm differs from ours, which focuses on cross-modal implicit learning rather than unimodal estimation, their results converge with ours, suggesting that cross-modal interactions are not fixed but shaped flexibly by task demands and sensory reliability. This further supports the idea that modal dominance emerges dynamically and that a common predictive system may underpin perceptual inference when one modality becomes more behaviorally informative. Complementary evidence by Rohe et al.^35^ shows that sensory reliability modulates perceptual inference through both weighting and structural adaptation of priors, suggesting that the brain flexibly adjusts its inferential strategy depending on the reliability of incoming signals. This is also confirmed by the results of the comparison between the models, which showed that the WCTE model produced results almost equivalent to the segregation models in both tasks. When one modality dominates in terms of sensory reliability, weighted integration can become functionally indistinguishable from unisensory inference. Thus, it would be interesting in future studies to manipulate the relative reliability of the two sensory modality (e.g., by degrading the dominant modality or enhancing the weaker one) to test whether such changes shift the integration strategy from winner-takes-all mechanism – observed here due to the strong sensory imbalance - toward more balanced Bayesian fusion. In such a case, we might expect the central tendency model (CTE) to better capture the behavioral data.

While these conjectures will need further investigation, what can be concluded based on the data reported here is that, at the behavioral and computational level, sensory context is crucial in shaping perception and takes priority over a generalized central tendency effect.

## Materials and methods

### Participants

Twenty-one participants (age: M = 20.48 years, S.D. = 1.64 years) with normal or corrected-to-normal vision and self-reported normal hearing participated in the study. Before starting the experiment, all participants provided written informed consent. The study protocol was approved by the University of Sydney Human Research Ethics Committee (HREC 2021/048), and all methods were performed following the relevant guidelines and regulations. Two participants were outliers in the time estimation task, and one in the spatial estimation task. Therefore, we remain with a sample of 19 participants for the temporal tasks and 20 participants for spatial tasks (see data analysis section).

### Stimuli and apparatus

The experiment was performed in a dark, sound-attenuated room. All stimuli were created and controlled in MATLAB (MathWorks, Natick, MA) with Psychtoolbox^36^. All visual stimuli were projected on a white sheet subtending ~ 65° in width using a PROPixx projector running at 120 Hz. Auditory stimuli were played using an array of eleven speakers (Visaton F8SC) spaced at 5.5° intervals and arranged horizontally behind the sheet. The center of the sheet corresponds to the center of the speaker array and, in the rest of the text, will be referred to as position 0°. Negative values refer to the left side, and positive values to the right.

For the stimuli used in the temporal tasks, we replicated the stimuli used by Cicchini et al.^3^. Auditory stimuli were pure tones of 520 Hz with a duration of 20 ms (with transitions smoothed by raised cosine of 3 ms width). The visual stimuli were white disks of 3° diameter with 80% contrast displayed on a grey background for 17 ms.

For the spatial tasks, the sounds were white-noise bursts of 20 ms (with transitions smoothed by raised cosine of 3 ms width), while the visual stimuli were white disks of 3° diameter displayed with 80% contrast on a grey background for 17ms.

Participants sat 120 cm from the projector screen in all conditions and tasks.

### Procedure

Each participant underwent four tasks within two sessions: a spatial bisection, a temporal bisection, a temporal (duration) estimation, and a spatial (length) estimation. Both estimation tasks consisted of 3 sessions: one session in which only auditory stimuli were presented (audio), one in which there were only visual stimuli (vision), and one in which both visual and auditory stimuli were presented in random order (interleaved A-V). Session orders were randomized among participants and the experiment took place over two days and lasted around one hour each day^11^.

#### Temporal estimation (Fig. 1A)

Each trial started with a fixation cross located in the center of the screen and presented two consecutive stimuli (either circles or sounds) located at -5° and 5° and separated by a random interval duration. Participants had to reproduce the interval duration by pressing and holding the keyboard’s space bar for the appropriate amount of time. As mentioned above, the task was divided into three sessions: audio, vision, and interleaved A-V. A different range of nine interval durations was used for audition and vision so that each session’s average duration differed. For auditory stimuli, the mean duration was 670 ms (range 490 ms to 850 ms), and for visual stimuli, the mean duration was 940 ms (range 760 ms to 1120 ms). In the interleaved A-V session, auditory and visual stimuli are presented randomly and, therefore, had a mean duration of 805 ms. Although the auditory and visual stimuli had different duration ranges, they were partially overlapping and shared three durations: 760 ms, 805 ms, and 850 ms. The audio and vision sessions (baselines) are composed of 180 trials each, while the interleaved A-V consisted of 360 trials (180 visual and 180 auditory).

The durations used in the temporal estimation task were adapted from Cicchini et al.^3^, focusing on sub-second intervals to remain within a unified temporal processing regime. This choice avoids transitions across distinct timing mechanisms that are believed to operate at different scales, and focuses only on the timescale of milliseconds ^[see 37^.

#### Spatial estimation (Fig. 1B)

In each trial, two consecutive stimuli (either circles or sounds) were presented, separated by 500 ms and a random distance (changing on the horizontal plane) and participants had to reproduce the length (or distance) between the two stimuli. After the stimuli disappeared, a black rectangle (always 1°) appeared in a random location on the screen pointing left or right, and the participants had to adjust the length of the rectangle, using the right and left arrow keys, to match the distance separating the sounds/circles, pressing enter to submit their estimate and start the subsequent trial. This task was divided into three sessions: audio, vision, and interleaved A-V. Each sensory modality was associated with a specific range of six lengths, so the average length of each session differed. The auditory (audio session) lengths ranged from 22° to 50° with an average of 36°. The visual (vision session) lengths ranged from 5.5° to 33° with an average of 19.5°. The two sets of stimuli are combined randomly in the interleaved A-V session, making the session average equal to 28°. In addition, auditory and visual stimuli shared three lengths within the same session (22°, 28°, 33°). The audio and vision sessions (baselines) are composed of 120 trials each, while the interleaved A-V consisted of 240 trials (120 visual and 120 auditory).

Spatial distances were determined by the constraints of the speaker set-up, including the number and resolution of speakers, while ensuring a comparable number of stimulus levels to the temporal task.

### Data analysis

#### Behavioral data

For the estimation’s tasks, first, to remove any individual bias related to possible over or underestimation and keep data normalized for the analysis of central tendency, we subtracted from each perceived distance/duration the average perceived distance/duration for all the trials, averaging the central stimulus distance/duration between the two sessions and modalities.

Second, we calculated the best linear fit between the perceived responses and the real stimuli (durations or lengths) using the MATLAB toolbox “Curve fitting toolbox"^38^ and function fit(real, perceived, ‘poly1’) for each session (audio, vision). From the ‘*fit*’ function, we extracted the RMSE, providing an overall performance measure. We used this parameter to assess whether there were any participant outliers. For each task (temporal and spatial), we considered participants whose RMSE deviated more than ± 3 standard deviations from the group median as outliers. We had to exclude two participants from the temporal tasks because one was an outlier for both the auditory and visual sessions. Another was an outlier for the visual session. In the spatial estimation, we excluded one outlying participant from the auditory session.

To statistically test whether RMSE differed between sensory modalities for each task, we compared the RMSE using a paired two-tailed t-test (via “*stats*” version 4.3.2 package in R).Next, we calculated the best linear fits to the data as the first step in calculating the regression index (RI). This was done separately for auditory and visual stimuli (in the interleaved A-V condition, the auditory and visual trials were separated into different bins). The RI is the difference between the slope of the best linear fit and the equality line (unbiased performance in which x equals y). An RI of 0 means veridical performance, while RIs > 1 indicate regression toward the mean. At the RIs level, no participant was found to be an outlier. Therefore, participants with RI values close to 1 were not excluded from the analysis, as they did not meet the exclusion criteria either on the predefined RMSE-based or on the RI-based. Their inclusion reflects natural inter-individual variability in the strength of the central tendency effect, consistent with previous reports^39,40^.

We also used the y-intercept (beta) of the linear fit as a parameter to evaluate a possible interaction between sensory modalities in the interleaved A-V session. In case the dominant modality influenced the estimation in the specific task (audio for time, vision for space), we expected no difference in this modality between the interleaved A-V and unimodal sessions. In contrast, the intercept of the non-dominant modality (vision-time and audio-space) should be different between interleaved A-V and unimodal sessions in the direction of the dominant modality.

To statistically test the differences between sessions and modality, for each of the two parameters, RI and beta, we used a linear mixed-effects model (via the “*lme4*” 1.1–21 package in R), using REML as convergence criteria. The model’s fixed effects were the within-participant “Session” (unimodal and interleaved A-V) and “Modality” (audio and vision), while the participant was added as a random effect^41^.

For all linear mixed-models, as a follow-up analysis, we estimated the model’s marginal means for factor combinations to investigate the significance of the linear mixed-model factors (via “emmeans” version 1.10.0 package in R). This analysis allowed us to compute and contrast the predicted probability distributions relative to each response level. We considered pairwise comparisons with *p* < 0.05 significant only after applying a family-wise error rate correction (FWER) according to the Holm-Bonferroni correction for multiple comparisons. Moreover, the degrees of freedom for post hoc were estimated using the Kenward–Roger approximation as implemented in the “emmeans” package in R. The linear mixed-effects model was run separately for the temporal and spatial estimations.

As a final step, we calculated sensory precision (SP) for both modalities in performing the estimation tasks, which will be useful for Bayesian models (see next paragraph). We perform this analysis only for the baseline sessions (only-audio and only-vision), because in these sessions it is possible to isolate purely sensory variability, whereas in the interleaved A-V session, the response is influenced by modality interactions. Nevertheless, even in baseline sessions, the SP could be masked by the central tendency effect; we applied the method used by Aston et al.^42^ to obtain the sensory precision net of the central tendency effect. To do so, for each participant, task, and modality, we fitted a linear regression between the presented stimulus values (either duration or length) and the corresponding responses, obtaining a slope. We then calculated the residuals, i.e., the difference between the observed and predicted responses from the linear fit. For each stimulus, we calculated the variance of the residuals, and since a slope different from 1 can inflate or deflate this variance, we corrected it by dividing the residual variance by the square of the slope, following the approach described by Aston et al.^42^. This provided a stimulus-by-stimulus estimate of sensory precision that was used in downstream modeling to assess how sensory noise scales with the physical stimulus range.

By calculating these values, we were also able to calculate a measure of relative reliability of the two modalities within each task, i.e., perceptual weight.

$$\:{{\upomega\:}}_{A}=\frac{{\sigma\:}_{V}^{2}}{({\sigma\:}_{A}^{2}+{\sigma\:}_{V}^{2})\:}\:;{{\upomega\:}}_{V}=1-\:{{\upomega\:}}_{A}\:\:$$

Subsequently, these weights were used to construct the weighted prior in the WCTE Bayesian model (see the Bayesian Models section for more details).

#### Bayesian models

To test whether our perception would be influenced by the central tendency effect or by the prior represented by the distribution mean of the dominant modality for that specific perceptual domain, we considered four models assuming that an ideal Bayesian observer drives perception.

Within the Bayesian framework, the estimated stimulus (or posterior) is the combination of two sources of information: the *likelihood*, i.e., the current noisy estimate of stimulus durations/lengths represented by a Gaussian distribution center at the current stimulus to estimate, and the *prior*, i.e., described by a Gaussian probability distribution function and centered on the average of the stimuli set.

In each model, the *likelihood* distribution would have a mean corresponding to the stimulus length/duration to be estimated (µ_L_ = S_i_) and a standard deviation (σ_L_) depending on the sensory sensitivity that we obtained from the calculation of sensory precision (SP) in the baseline sessions for each stimulus (σ_L_(S_i_) = SP_A_(S_i_) = σ_A_(S_i_) or σ_L_(S_i_) = SP_V_(S_i_)= σ_V_(S_i_)). While according to the Bayesian rule, the posterior is a Gaussian centered at:

1 $$\:{{\upmu\:}}_{R}={{\upmu\:}}_{L}-\frac{{\sigma\:}_{L}{\left({S}_{i}\right)}^{2}\left({{\upmu\:}}_{L}-\:{{\upmu\:}}_{P}\right)}{({\sigma\:}_{L}{\left({S}_{i}\right)}^{2}+{\sigma\:}_{P}^{2})\:}\:\:$$

Formula [1] can be rewritten as

2 $$\:{{\upmu\:}}_{R}\left({S}_{i}\right)={{\upmu\:}}_{L}\left({S}_{i}\right)\left(1-\frac{{\sigma\:}_{L}{\left({S}_{i}\right)}^{2}}{{\sigma\:}_{L}{\left({S}_{i}\right)}^{2}+{\sigma\:}_{P}^{2}}\right)+{{\upmu\:}}_{P}\left(\frac{{\sigma\:}_{L}{\left({S}_{i}\right)}^{2}}{{\sigma\:}_{L}{\left({S}_{i}\right)}^{2}+{\sigma\:}_{P}^{2}}\right)\:\:$$

And summarized as

3 $$\:{{\upmu\:}}_{R}={{\upmu\:}}_{L}{{\upomega\:}}_{L}\left({S}_{i}\right)+\:{{\upmu\:}}_{P}{{\upomega\:}}_{P}\left({S}_{i}\right)\:\:$$

Where ω_L_ (S_i_)is the weight assigned to the likelihood, and ω_P_ is the weight assigned to the prior.

4 $$\:{{\upomega\:}}_{P}\left({S}_{i}\right)=\frac{{\sigma\:}_{L}{\left({S}_{i}\right)}^{2}}{({\sigma\:}_{L}{\left({S}_{i}\right)}^{2}+{\sigma\:}_{P}^{2})\:}\:\:$$

5 $$\:{{\upomega\:}}_{L}\left({S}_{i}\right)=\frac{{\sigma\:}_{P}^{2}}{({\sigma\:}_{L}{\left({S}_{i}\right)}^{2}+{\sigma\:}_{P}^{2})\:}\:\:$$

What we manipulated is the nature of the *prior* distribution. For each of the models tested, we modeled the prior as a truncated normal distribution. This approach considers the fact that stimuli are limited, which leads to a mismatch if using a full Gaussian. To estimate the parameters of the truncated normal prior, we solved the following system of equations for each condition:

6 $$\:E\left[X\right]={\upmu\:}+{\upsigma\:}\:.\:\frac{\varphi\:\left({\upalpha\:}\right)-\varphi\:\left(\beta\:\right)}{\varPhi\:\left(\beta\:\right)-\varPhi\:\left({\upalpha\:}\right)}$$

7 $$\:Var\left[X\right]={\sigma\:}^{2}\left[1+\frac{\alpha\:\varphi\:\left({\upalpha\:}\right)-\beta\:\varphi\:\left(\beta\:\right)}{\varPhi\:\left(\beta\:\right)-\varPhi\:\left({\upalpha\:}\right)}-\:{\left(\:\frac{\varphi\:\left({\upalpha\:}\right)-\varphi\:\left(\beta\:\right)}{\varPhi\:\left(\beta\:\right)-\varPhi\:\left({\upalpha\:}\right)}\right)}^{2}\right]$$

Where $\:\alpha\:=\frac{\text{a}-{\upmu\:}}{\sigma\:}$ and $\:\beta\:=\frac{\text{b}-{\upmu\:}}{\sigma\:}$, and *a*, *b* are the lower and upper bounds of the stimulus distribution associated to the theoretical prior. These equations were solved numerically using constrained nonlinear optimization (MATLAB’s lsqnonlin) to match the empirical mean and standard deviation of the stimulus set, thus obtaining a more realistic estimation of the prior’s mean and variance.

In the segregated model (Fig. 6 first and second column), we assumed that the *prior* distribution is sensory-dependent. In this case, to calculate the mean (µ_P_) and standard deviation (σ_P_) of the *prior* distribution using the truncated normal distribution we used the set of lengths/durations associated with the sensory modality for each task. Thus, based on whether we consider the model auditory segregated (SegAudio – Fig. 6A) or visual segregated (SegVision – Fig. 6B), the formula [2] can be rearranged as

8 $$\:{{\upmu\:}}_{R}={S}_{i}\left(1-\frac{{\sigma\:}_{A}^{2}}{{\sigma\:}_{A}^{2}+{\sigma\:}_{\text{s}\text{d}\text{A}}^{2}}\right)+{{\upmu\:}}_{A|V}\left(\frac{{\sigma\:}_{A}^{2}}{{\sigma\:}_{A}^{2}+{\sigma\:}_{\text{s}\text{d}\text{A}}^{2}}\right)\:\:$$

for the SegAudio, and the SegVision as

9 $$\:{{\upmu\:}}_{R}={S}_{i}\left(1-\frac{{\sigma\:}_{V}^{2}}{{\sigma\:}_{V}^{2}+{\sigma\:}_{\text{s}\text{d}\text{V}}^{2}}\right)+{{\upmu\:}}_{A|V}\left(\frac{{\sigma\:}_{V}^{2}}{{\sigma\:}_{V}^{2}+{\sigma\:}_{\text{s}\text{d}\text{V}}^{2}}\right)\:\:$$

In the central tendency model (CTE – Fig. 6C), we assumed that the *prior* distribution is average dependent. In this case, the mean (µ_GM_) and the standard deviation (σ_GM_) of the *prior* distribution were derived from the full stimulus range, independently from the sensory modality, using the truncated normal distribution. Formula [2] can be rearranged as

10 $$\:{{\upmu\:}}_{R}={S}_{V|A}\left(1-\frac{{\sigma\:}_{V|A}^{2}}{{\sigma\:}_{V|A}^{2}+{\sigma\:}_{\text{s}\text{d}\text{V}|\text{s}\text{d}\text{A}}^{2}}\right)+{{\upmu\:}}_{GM}\left(\frac{{\sigma\:}_{V|A}^{2}}{{\sigma\:}_{V|A}^{2}+{\sigma\:}_{\text{s}\text{d}\text{V}|\text{s}\text{d}\text{A}}^{2}}\right)\:\:$$

For the last model, the weighted central tendency model (WCTE – Fig. 6D), we assumed that the *prior* distribution varies depending on the reliability of the sensory modality. In this case, the mean of the *prior* distribution (µ_W_) is calibrated according to how much weight is given to each sensory modality. If, for example, visual information is more reliable than auditory information, then µ_W_ will be similar to the mean of the set of lengths/durations associated with the sensory modality (µ_V_) or vice versa. In contrast, the standard deviation of the prior was computed as the square root of the weighted sum of variances of the truncated priors associated with each modality of the segregated models.

Thus, formula [2] can be rearranged as

11 $$\:{{\upmu\:}}_{R}={S}_{V|A}\left(1-\frac{{\sigma\:}_{V|A}^{2}}{{\sigma\:}_{V|A}^{2}+{\sigma\:}_{\text{s}\text{d}\text{V}|\text{s}\text{d}\text{A}}^{2}}\right)+{{\upmu\:}}_{w}\left(\frac{{\sigma\:}_{V|A}^{2}}{{\sigma\:}_{V|A}^{2}+{\sigma\:}_{\text{s}\text{d}\text{V}|\text{s}\text{d}\text{A}}^{2}}\right)\:\:$$

We can express µ_W_ as

12 $$\:{{\upmu\:}}_{w}={{\upmu\:}}_{A}{{\upomega\:}}_{A}+\:{{\upmu\:}}_{V}{{\upomega\:}}_{V}\:\:$$

Each prediction was calculated for each participant based on individual sensitivity to the task and sensory modality.

It is important to note that, although we implemented separate segregation models for auditory and visual priors, we did not include a fifth model in which each trial would be fitted using the prior of the same sensory modality (i.e., auditory prior for auditory stimuli, visual prior for visual stimuli). Such a hybrid model, representing complete sensory segregation, would provide a useful theoretical basis for assessing complete independence between the two modalities without any cross-modal effect. Although this logic is indirectly reflected in our individual segregation models, future work could explicitly test this combined approach within a unified framework.

Finally, in order to evaluate which simulation of the data explains the experimental data better, we run a linear mixed-effects model for each Bayesian model, using the real data as a dependent variable, and the model’s fixed effects were “Stimulus” (durations/lengths) and “Simulated-data”. At the same time, the participant was added as a random effect. We used the Bayesian Information Criterion (BIC) to determine the best-performing model (via *anova* function, “*stats*” version 4.3.2 package in R) for each task. The BIC favors models with a higher maximum log-likelihood, reflecting a better fit to the data, while penalizing those with more parameters, indicating greater complexity. For a given dataset, lower BIC values mean better model performance. Moreover, we calculated the R-square for the fitted models using the function “*R2*” (via “*semEff*” version 0.6.1 package in R) and set the correlations as Pearson. This function also allows the ‘predicted R-squared’ to be obtained, calculated via the same formula as for R-squared but using cross-validated, rather than original, fitted values.

## Data Availability

The datasets analyzed during the current study are available in the Zenodo repository, at this link: https://doi.org/10.5281/zenodo.15240424.
